# Inhibition of FASN and ERα signalling during hyperglycaemia-induced matrix-specific EMT promotes breast cancer cell invasion via a caveolin-1-dependent mechanism

**DOI:** 10.1016/j.canlet.2018.01.028

**Published:** 2018-04-10

**Authors:** H.A. Zielinska, J.M.P. Holly, A. Bahl, C.M. Perks

**Affiliations:** a IGFs & Metabolic Endocrinology Group, School of Clinical Sciences, University of Bristol, Learning and Research Building, Southmead Hospital, Bristol BS10 5NB, UK; b Department of Clinical Oncology, Bristol Haematology and Oncology Centre, University Hospitals Bristol, Bristol, UK

**Keywords:** Breast cancer, Warburg effect, Hyperglycaemia, Epithelial to mesenchymal transition, Fibronectin, ECM, Extracellular matrix, EMT, Epithelial to mesenchymal transition, ERα, Estrogen receptor α, FASN, Fatty acid synthase, GFPT1, Glutamine–fructose-6 phosphateransaminase 1, GLUT, Glucose transporter, G6PD, Glucose-6-phosphate dehydrogenase, LDH, Lactate dehydrogenase, MCT, Monocarboxylate transporter, MET, Mesenchymal to epithelial transition, MMP, Matrix metalloproteinase, NS, Non-silencing, ns, not significant, OXPHOS, Oxidative phosphorylation, PARP, Poly(ADP-ribose) polymerase, PHGDH, Phosphoglycerate dehydrogenase, TALDO1, Transaldolase 1, TCA, tricarboxylic acid, TGFβ, Transforming growth factor β, TKT, Transketolase

## Abstract

Since disturbed metabolic conditions such as obesity and diabetes can be critical determinants of breast cancer progression and therapeutic failure, we aimed to determine the mechanism responsible for their pro-oncogenic effects. Using non-invasive, epithelial-like ERα-positive MCF-7 and T47D human breast cancer cells we found that hyperglycaemia induced epithelial to mesenchymal transition (EMT), a key programme responsible for the development of metastatic disease. This was demonstrated by loss of the epithelial marker E-cadherin together with increases in mesenchymal markers such as vimentin, fibronectin and the transcription factor SLUG, together with an enhancement of cell growth and invasion. These phenotypic changes were only observed with cells grown on fibronectin and not with those plated on collagen. Analyzing metabolic parameters, we found that hyperglycaemia-induced, matrix-specific EMT promoted the Warburg effect by upregulating glucose uptake, lactate release and specific glycolytic enzymes and transporters. We showed that silencing of fatty acid synthase (FASN) and the downstream ERα, which we showed previously to mediate hyperglycaemia-induced chemoresistance in these cells, resulted in suppression of cell growth: however, this also resulted in a dramatic enhancement of cell invasion and SLUG mRNA levels via a novel caveolin-1-dependent mechanism.

## Highlights

• Exposure to hyperglycaemia and fibronectin induced EMT and promoted the Warburg effect in ERα-positive breast cancer cells.
• Inhibition of the FASN/ERα signalling pathway resulted in a dramatic enhancement of cell invasion.
• Caveolin-1 mediated the pro-invasive phenotype triggered by targeting FASN or the ERα.

## 1 Introduction

Evidence suggests that breast cancer patients presenting with metabolic conditions such as obesity and diabetes do not respond as well to treatment and have a higher overall mortality rate: the major influence being hyperglycaemia [[1], [2], [3]]. Otto Warburg in the 1920s [4,5] identified a distinct metabolic profile of cancer cells characterised by increased glycolytic activity and lactate production even in the presence of oxygen. The so-called Warburg effect plays a crucial role in sustaining the increased bioenergetic and biosynthetic requirements of rapidly proliferating cancer cells and has been the subject of intense investigations in recent years [6,7]. Despite the resurgence in interest in cancer cell metabolism and its recognition as one of the fundamental hallmarks of tumours, the development of effective metabolic interventions to prevent breast cancer progression remains a continuing challenge.

Death from breast cancer is generally due to the development of metastatic disease. As many as 30% of patients with early breast cancer develop metastatic disease with the majority of these being resistant to current therapies [8]. The metastatic cascade is a complex multistage event that involves the detachment of cancer cells from the primary tumour, entry into the blood or lymphatic system and invasion into a new microenvironment that is conducive to their attachment and subsequent growth. Changes in the tumour microenvironment including extensive remodelling of extracellular matrix (ECM) and alterations in the surrounding stromal cells are strongly associated with malignant transformation [9,10]. For instance, fibronectin -a key structural component of the ECM has been heavily implicated in the process of tumour development, with reports linking elevated fibronectin expression in breast cancer stroma with tumour aggressiveness and poorer prognosis [11,12]. The loss of epithelial differentiation referred to as epithelial to mesenchymal transition (EMT) has been proposed to contribute to metastatic progression. The downregulation of epithelial markers (such as E-cadherin), increases in mesenchymal markers (such as N-cadherin, vimentin and fibronectin) and transcription factors (including SNAIL and SLUG) together with the acquisition of increased motility, invasion and stem cell properties are key defining features of the EMT program [13]. While it is overwhelmingly clear that EMT plays a critical role in cancer progression and metastasis only a few studies have investigated the impact of the metabolic status on this process. As such, we wished to explore the effects of metabolic conditions on EMT with a view to identifying potential novel targets for intervention.

In this study, we report that exposure of the estrogen receptor α (ERα)-positive MCF-7 and T47D human breast cancer cells to high glucose levels induced EMT; however, this was only observed with cells grown on fibronectin but not with those plated on the basement membrane component collagen. Furthermore, we show that hyperglycaemia-induced, matrix-specific EMT triggered a metabolic switch towards the Warburg effect by upregulating glucose uptake, lactate release and specific glycolytic enzymes and transporters. We also attempted to block the hyperglycaemia-induced EMT phenotype by targeting the lipogenic enzyme fatty acid synthase (FASN) and the downstream ERα which, as previously shown by us, cooperate together to mediate hyperglycaemia-induced chemoresistance in these cells [14,15]. We found that whilst targeting FASN or ERα signalling effectively suppressed cell growth, it also resulted in enhanced invasive capacity. Mechanistically, these pro-invasive effects were found to be driven by caveolin-1, a lipid raft protein implicated in various aspects of tumour progression including EMT [[16], [17], [18]]. Overall, these novel findings highlight the significance of the metabolic status as a powerful factor that determines which women have cancers that progress to life-threatening metastatic disease. Furthermore, we reveal the pro-invasive consequences of targeted inhibition of FASN and ERα signalling in ERα-positive breast cancers and we provide evidence for the therapeutic potential of caveolin-1 in this specific context.

## 2 Materials and methods

### 2.1 Cell culture

The human breast cell lines MCF-7, T47-D, MCF10A, ZR-75-1, MDA-MB-231 and Hs578T were purchased from the American Type Culture Collection (ATCC, Molsheim, France). All cell lines were cultured as described previously [14,19]. For all experiments, cells were seeded onto fibronectin or collagen-coated 6-well plates at a density of 0.1 × 10^6^/well in 5 mM glucose-containing DMEM growth media for 24 h and then switched to serum free media containing different concentrations of glucose including 5 mM (normal, Sigma: D5546), 9 (prepared from 5 and 25 mM glucose-containing DMEM) and 25 mM (high glucose, Sigma: D6429) for 48 h in the presence or absence of target siRNA to the ERα, FASN, caveolin-1 or a non-silencing siRNA or were treated with anti-estrogens fulvestrant or tamoxifen as outlined before [15]. We used two different siRNAs to silence ERα, FASN and caveolin-1; we confirmed the effective knockdown of caveolin-1 using a second siRNA sequence in the present study (data not shown). All siRNAs were purchased from Qiagen and their sequences were as follows: 5′-CUGACACUUUAAUUACCAATT-3′ and 5′-UUGGUAAUUAAAGUGUCAGGA-3′ for caveolin-1 siRNA 1, 5′-CAGUCUUCCUGACACUUUATT-3′ and 5′-UAAAGUGUCAGGAAGACUGGA-3′ for caveolin-1 siRNA 2. Fibronectin-coated 6-well plates were purchased from Greiner Bio-One. Collagen-coated 6-well plates were prepared according to the manufacturer's protocol (Sigma).

### 2.2 Cell growth

Cell growth was assessed via direct count of viable cells in a haemocytometer using trypan blue as described previously [14]. Changes in cell growth were also confirmed by assessing the protein abundance of a cell cycle protein cyclin D1 using western immunoblotting.

### 2.3 Transwell invasion assay

Cells were seeded onto collagen or fibronectin-coated dishes as described above. 48 h post-treatment, cells were trypsinised as described previously [14] and 5 × 10^5^ cells were seeded into matrigel pre-coated 8 μm pore transwell inserts (Milipore) in the corresponding glucose-containing serum free media and allowed to invade towards medium containing 10% FBS for 24 h. After incubation, cells in the insert (non-invaded cells) were removed with a cotton swab and invaded cells were fixed and stained with DAPI. The number of invaded cells was manually quantified in 5 randomly selected fields of view under ×20 magnification.

### 2.4 Western immunoblotting

Western blot analysis was performed as described previously [14]. Briefly, 20 μg of protein were run on 10% SDS-PAGE, transferred to nitrocellulose membrane (BioRad) and immunoblotted with the following antibodies: fibronectin (1:500, BD Biosciences), E-cadherin (1:1000, Cell Signalling), vimentin (1:500, BD Biosciences), N-cadherin (1:1000, BD Biosciences), FASN (1:1000, BD Biosciences), ERα (1:750, Santa Cruz), cyclin D1 (1:1000, Santa Cruz), PARP (1:1000, BD Biosciences), caveolin-1 (1:500, Santa Cruz), GAPDH (1:5000, Millipore) and tubulin (1:5000, Millipore), following the manufacturer's instructions. After incubation with specific secondary antibodies conjugated to peroxidase (Sigma), proteins were visualised by Clarity ECL substrate (BioRad) using BioRad Chemidoc XRS + system and analysed using Image lab software (BioRad).

### 2.5 Glucose uptake and lactate and citrate measurements

After treatments, the cells were washed with PBS and incubated in Krebs-Ringer bicarbonate (KRB) buffer for 15 min at 37 °C followed by the addition of the non-metabolisable glucose analogue tritiated 2-deoxyglucose (0.5 μCi per well, PerkinElmer) for 5 min. Subsequently, the cells were washed three times with ice-cold PBS and then solubilised in 1% (v/v) Triton X-100 in PBS. The incorporated radioactivity was assessed using a Beckman Scintillation Counter LS6500. Data were recorded as disintegrations per minute (DPM). Lactate and citrate levels were measured in cell supernatants using colorimetric Lactate Assay and Citrate Assay Kits (BioVision) following the manufacturer's instructions and the absorbance was measured using iMark Microplate Absorbance Reader (BioRad). Data were normalised to protein content.

### 2.6 qPCR and primers

Total RNA was extracted with Ribozol RNA extraction reagent (Amresco). One μg of RNA was reverse-transcribed using the high capacity RNA-to-cDNA kit (Life Technologies). qPCR was performed with SYBR Green JumpStart Tag ReadyMix (Sigma) according to the manufacturer's protocol using ABI StepOne Plus Real-Time PCR System (Applied Biosystems, 4376600). Relative mRNA levels were determined using 2^−ΔCt^ method. The *GAPDH* gene was used as an internal control. The primer sequences were taken from Kondaveeti et al. [20] and their specificity confirmed by melting curve analysis (data not shown). All primer pairs were synthesised by Sigma.

### 2.7 Statistical analysis

Data were analysed with SPSS 12.0.1 for Windows using one-way ANOVA followed by least significant difference (LSD) post-hoc test. A statistically significant difference was considered to be present at p < .05.

## 3 Results

### 3.1 Hyperglycaemia induces EMT in breast cancer cells grown on a fibronectin substrate

**Fig. 1:**
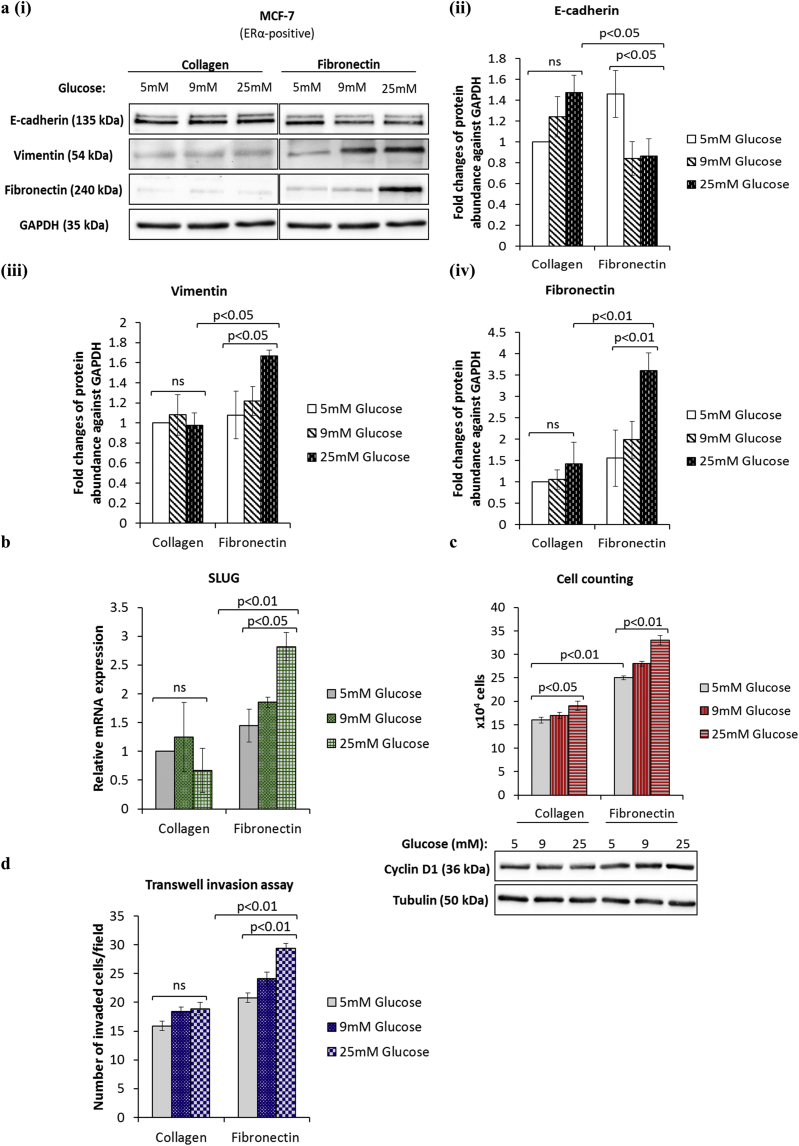
**Exposure of breast cancer cells to hyperglycaemia and fibronectin induces EMT**. **(a, i)** MCF-7 cells were cultured as described in Materials and methods section. Western blotting was performed to examine the protein abundance of the indicated EMT markers. **(a, ii)** Densitometry was performed to quantify the protein levels of EMT markers. **(b)** SYBR green-based qPCR analysis of SLUG mRNA levels. **(c)** Changes in cell growth were assessed by direct count of viable cells in a haemocytometer (upper panel) and assessment of cyclin D1 protein expression by Western blotting (lower panel). **(d)** Cell invasion was measured using the transwell assay. In all cases, results shown are representative of three independent experiments, each performed in triplicate and are expressed as means of ±SEM. (For interpretation of the references to color in this figure legend, the reader is referred to the Web version of this article.)

**Supplementary Fig. S1:**
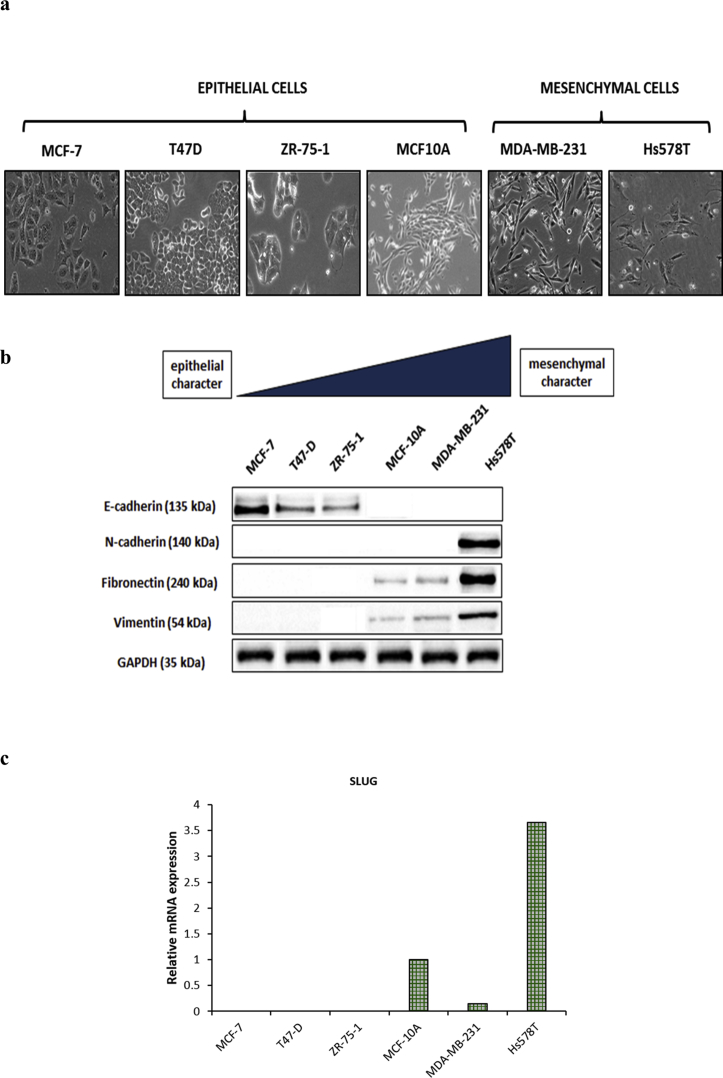
**Analysis of EMT features in a panel of breast cancer cell lines and non-transformed human mammary cells**. **(a)** Morphology of epithelial and mesenchymal cells assessed under phase contrast light microscopy. Epithelial cells are characterised by cobblestone-like morphology whereas mesenchymal cells display spindle-like, elongated phenotype. **(b)** Western blot analysis of EMT markers. Decreased expression of epithelial phenotype marker E-cadherin and enhanced expression of mesenchymal markers N-cadherin, fibronectin and vimentin were found in parallel to morphological features associated with epithelial and mesenchymal phenotypes. GAPDH was used as a loading control. **(c)** The mRNA levels of the EMT transcription factor SLUG were quantified using SYBR green-based qPCR in the indicated cell lines. MCF-10A (non-malignant), MCF-7, ZR-75-1 and T47-D (non-invasive ERα-positive), MDA-MB-231 and Hs578T (highly invasive ERα-negative) cells.

**Supplementary Fig. S2:**
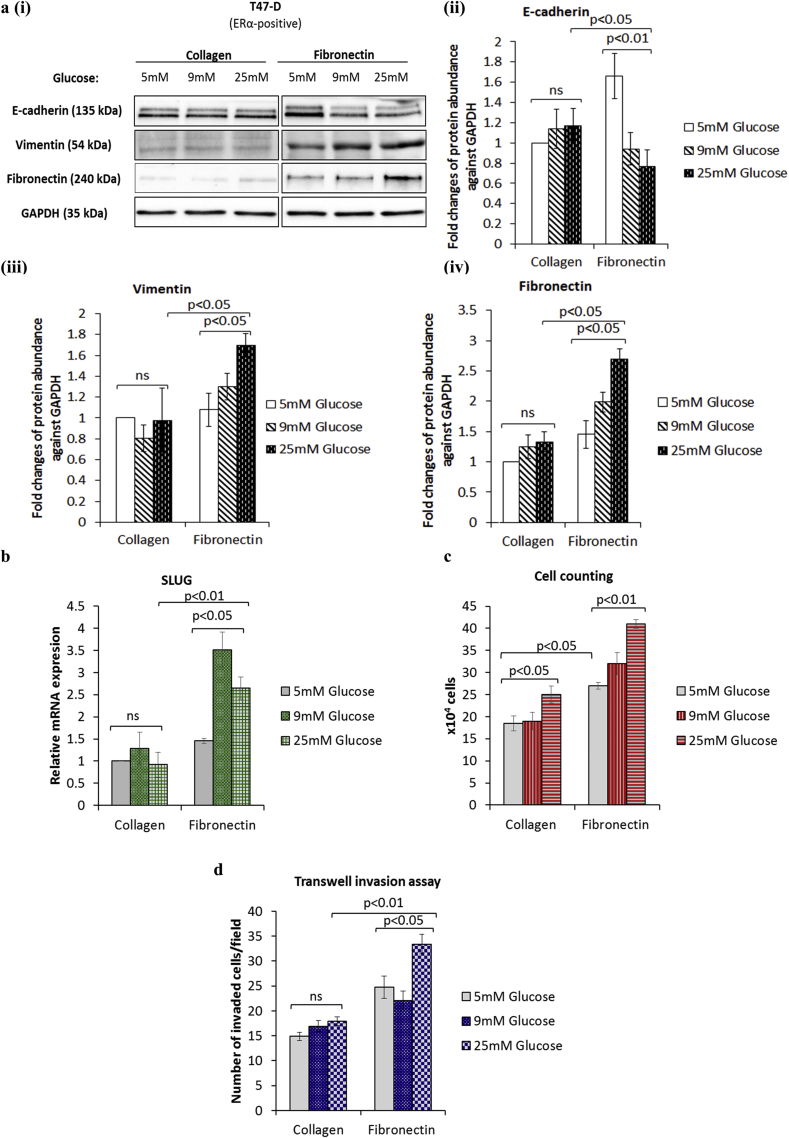
**Validation of hyperglycaemia-induced, matrix-specific EMT phenotype in another epithelial breast cancer cell line**. T47-D cells were seeded and analysed as described in the legend of Fig. 1. In all cases, results shown are representative of three independent experiments, each performed in triplicate and are expressed as means of ±SEM.

**Supplementary Fig. S3:**
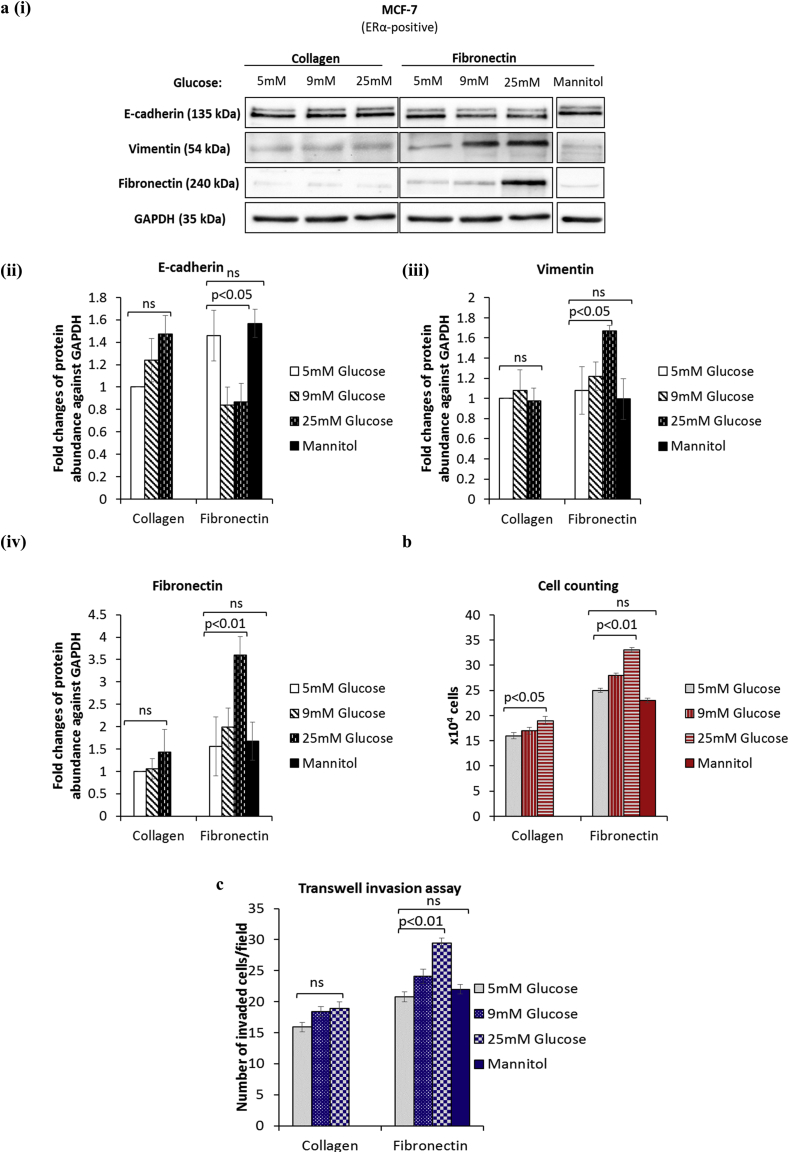
**Hyperglycaemia-induced, matrix-specific EMT phenotypic properties are not confounded by osmolarity**. Changes to the hyperglycaemia-induced, matrix-specific EMT phenotype following incubation of MCF-7 cells with an osmotic control (5 mM glucose supplemented to 25 mM with mannitol) medium were assessed by (a) western blot analysis of EMT markers, (b) direct count of viable cells in a hemocytometer and (c) transwell invasion assay. In all cases, results shown are representative of three independent experiments, each performed in triplicate and are expressed as means of ±SEM.

To explore the effects of metabolic conditions on EMT we tested three concentrations of glucose: 5 mM glucose (euglycaemic), 9 mM glucose (levels observed in a reasonably controlled diabetic patient) and 25 mM glucose (hyperglycaemic). A localised breast cancer predominantly interacts with collagen in the basement membrane but once the cancer has spread it is increasingly exposed to fibronectin in the adjacent stroma [21]. To mimic these stages of cancer progression we analysed the effects of exposure to different levels of glucose on cells grown on collagen and fibronectin. Having performed the phenotypic characterisation of EMT status in various breast cancer cell lines (Supplementary Fig. S1) we chose MCF-7 and T47-D cells that exhibited predominantly epithelial characteristics, in all subsequent experiments. Our results showed that increasing levels of glucose were associated with the promotion of EMT only in cells cultured on fibronectin and not with those plated onto collagen. From the lowest to the highest concentration of glucose we observed significant reductions in E-cadherin (p < .05) and significant increases in fibronectin (p < .01), vimentin (p < .05) and the transcription factor SLUG (p < .05) (Fig. 1a (i-iv) and b). We next examined changes in cell phenotype such as growth and invasion. As shown in Fig. 1c (upper panel), hyperglycaemia induced a dose-dependent increase in cell growth on both matrices but this was more marked with cells on fibronectin (1.1 (p < .05) and 1.3 (p < .01) fold increases from 5 to 25 mM glucose on collagen and fibronectin). The level of growth observed at every glucose concentration was significantly greater with cells exposed to fibronectin in comparison to those on collagen (e.g. 1.6-fold increase at 5 mM glucose; p < .01). Consistent with these results, the abundance of the cell cycle protein cyclin D1 mirrored these changes in growth (Fig. 1c, lower panel). We also showed that on collagen the level of invasion remained the same regardless of glucose concentrations, whereas on fibronectin invasion increased in a dose-dependent manner (1.4-fold increase from 5 to 25 mM glucose; p < .01; Fig. 1d). We obtained similar results using another non-invasive epithelial cell line, T47D (Supplementary Fig. S2). We used uncoated plastic plates as an additional control and we observed that there was no significant difference in the levels of EMT markers, cell growth and invasion between cells grown on collagen-coated and uncoated plates (data not shown). We also confirmed that an osmotic control medium (5 mM glucose supplemented to 25 mM with mannitol) did not have any effect on EMT phenotypic properties in MCF-7 cell line (Supplementary Fig. S3). These results suggest that exposure of breast cancer cells to hyperglycaemia and a more advanced tumour microenvironment such as fibronectin promotes EMT.

### 3.2 Hyperglycaemia-induced EMT phenotype is reversed upon silencing FASN

**Fig. 2:**
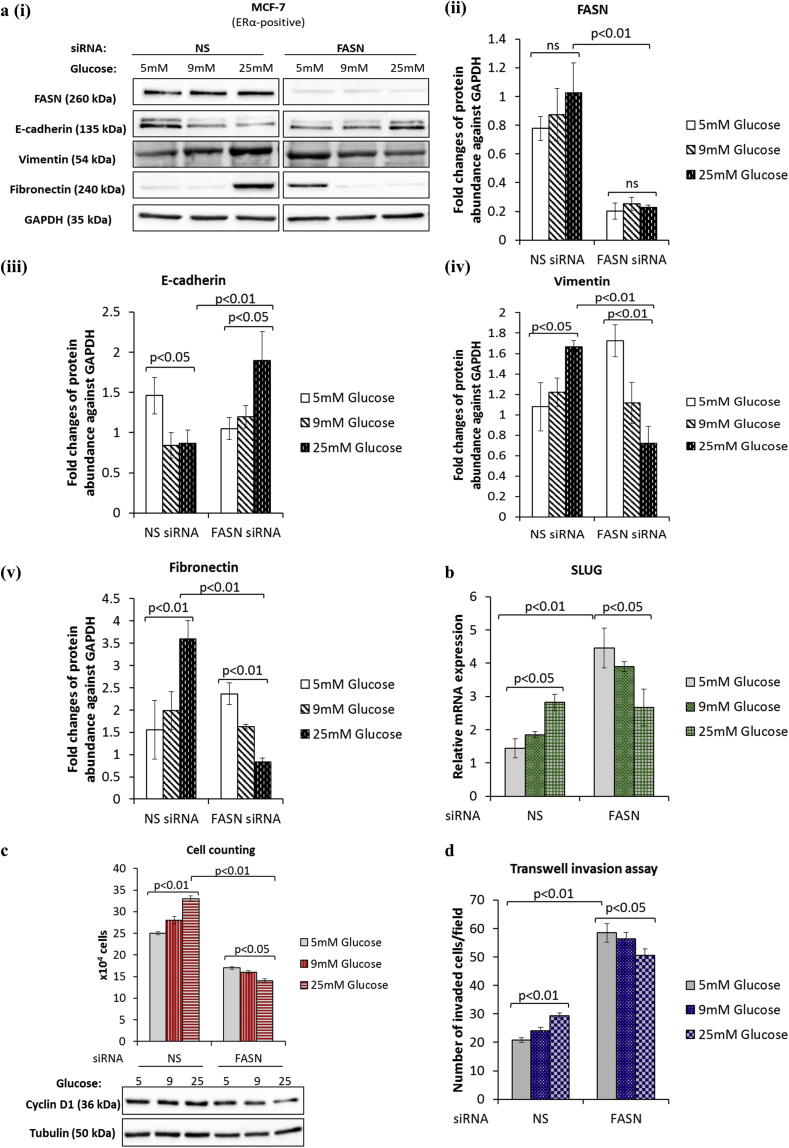
**Silencing of FASN results in the reversal of hyperglycaemia-induced, matrix-specific EMT phenotypic properties and enhanced cell invasion**. Changes to the hyperglycaemia-induced EMT phenotype following siRNA-mediated knockdown of FASN were assessed by **(a)** western blot analysis of EMT markers, **(b)** quantification of SLUG mRNA levels by qPCR, **(c)** cell counting and **(d)** transwell invasion assay. Results shown are representative of three separate experiments, each performed in triplicate. Data are represented as mean ± SEM. (NS = non-silencing).

FASN is a key enzyme responsible for the synthesis of fatty acids and a number of clinical reports link its expression with more aggressive tumour characteristics and worse clinical outcomes [22]. Our earlier work in the MCF-7 and T47D cells identified FASN as a key mediator of hyperglycaemia-induced chemoresistance [14]. We speculated that FASN might be a likely candidate in mediating the hyperglycaemia-induced, matrix-specific EMT observed in our cell model. To explore this, we performed siRNA-mediated knock down of FASN and assessed its impact on EMT markers, cell growth and invasion. We did not observe any significant change in the protein levels of FASN during hyperglycaemia-induced, matrix-specific EMT with cells grown on fibronectin. Whilst we cannot rule out an increase in FASN enzyme activity we previously found that inhibiting FASN signalling by either siRNA or through administration of C75, an inhibitor of FASN activity, had the same effect [14]. Examining the expression of EMT markers, we found that silencing FASN reversed the hyperglycaemia-induced EMT phenotype leading to increased expression of E-cadherin (p < .05) and decreased vimentin (p < .01) and fibronectin (p < .01) (Fig. 2a (i-v)). These changes in EMT markers were still glucose-dependent but in the opposing direction when FASN expression was silenced. The changes in mRNA levels for SLUG were not only dose-dependently reversed with respect to glucose but also greatly upregulated in FASN silenced cells when compared to the non-silencing control (3-fold increase at 5 mM glucose with FASN knocked-down; p < .01; Fig. 2b). While FASN knock-down resulted in the suppression of cell growth and a dose-dependent reversal in the effects of glucose on EMT markers, we observed a dramatic stimulation of cell invasion at every glucose concentration (e.g. 2.8-fold increase in invasion at 5 mM glucose with FASN knocked down; p < .01; Fig. 2d). Whilst these results suggest that FASN plays a key role in EMT and cell growth under hyperglycaemic conditions, as these were inhibited when FASN was silenced, our data also indicates that silencing FASN enhanced their invasive ability.

### 3.3 Effect of hyperglycaemia-induced, matrix-specific EMT on metabolic parameters

**Fig. 3:**
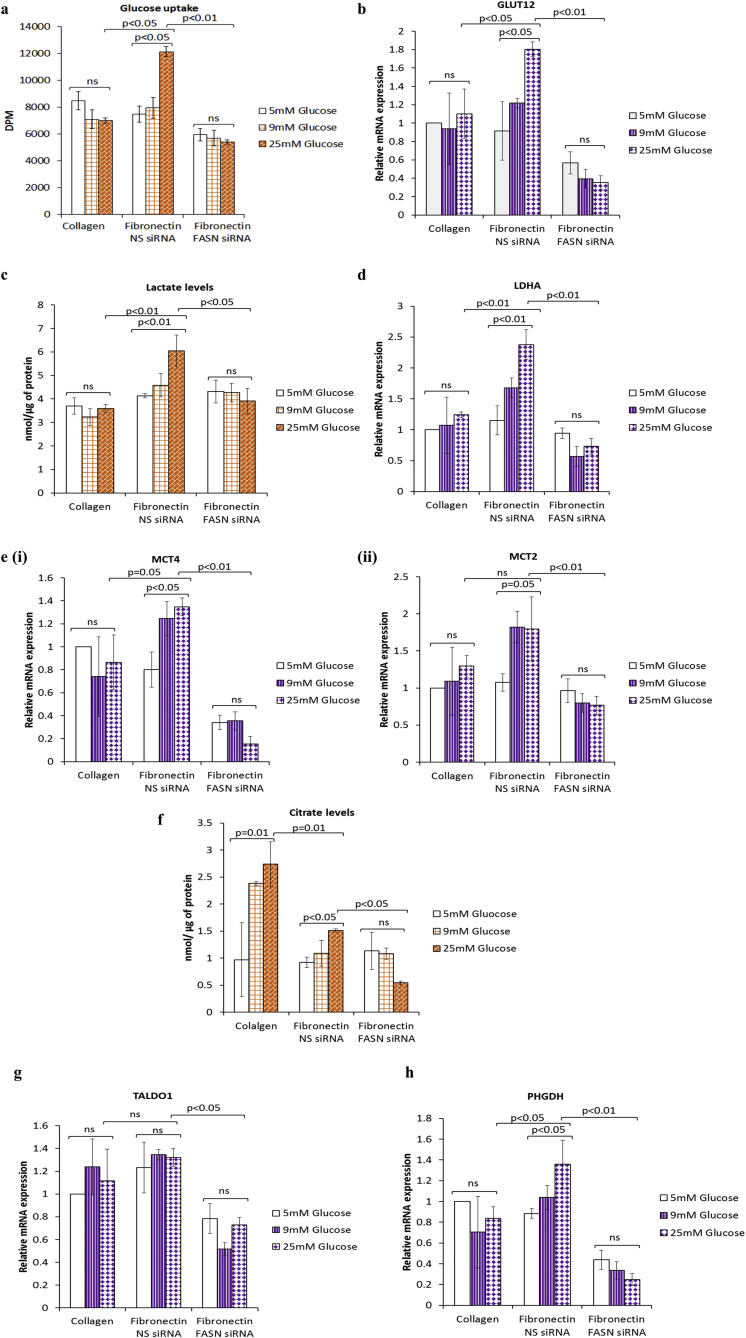
**Hyperglycaemia-induced, matrix-specific EMT triggers a metabolic switch towards the Warburg effect**. **(a**) Glucose uptake was assessed using the non-metabolisable glucose analogue, tritiated 2-deoxyglucose, in MCF-7 cells with or without FASN silenced with siRNA. The mRNA levels of the indicated metabolic enzymes were detected using SYBR green-based QPCR as shown in **b, d, e, g** and **h**. Lactate and citrate levels were measured in cell supernatants using colorimetric based assays **(c and f)**. In all cases, results shown are representative of three independent experiments, each performed in triplicate and are expressed as means of ±SEM. (For interpretation of the references to color in this figure legend, the reader is referred to the Web version of this article.)

**Supplementary Fig. S4:**
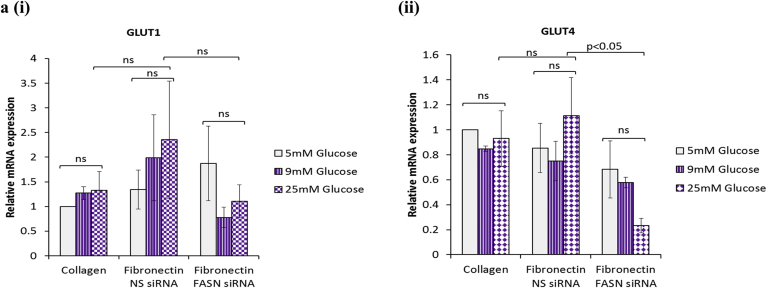
**GLUT1 and GLUT4 do not participate in hyperglycaemia-induced, matrix-specific EMT**. The mRNA levels of GLUT1 **(a, i)** and GLUT4 **(a, ii)** were quantified using SYBR green-based qPCR in MCF-7 cells cultured as described in Materials and methods section. Results shown are representative of three independent experiments, each performed in triplicate. Data are represented as mean ± SEM.

**Supplementary Fig. S5:**
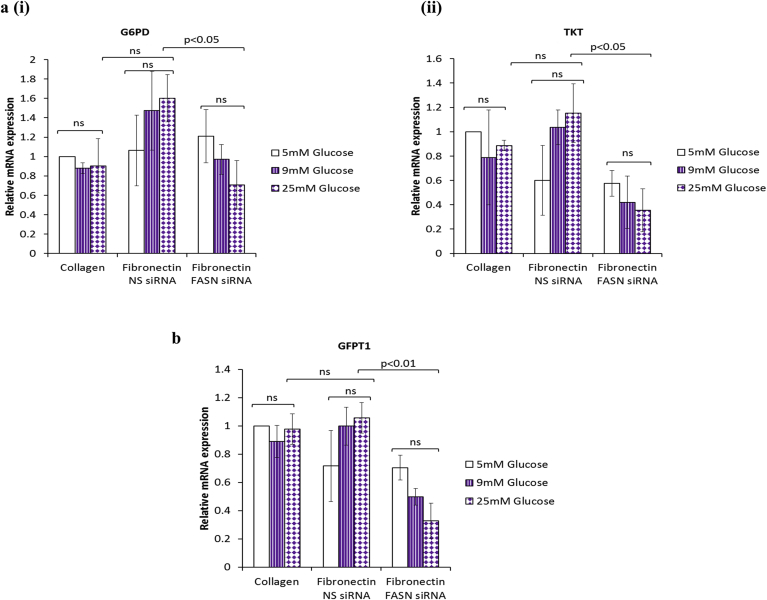
**Effect of hyperglycaemia-induced, matrix-specific EMT on the biosynthetic pathway genes**. The mRNA levels of G6PD, TKT **(a, i&ii)** and GFPT1 **(b)** were quantified using SYBR green-based qPCR in MCF-7 cells cultured as described in Materials and methods section. Results shown are representative of three independent experiments, each performed in triplicate. Data are represented as mean ± SEM.

We found that hyperglycaemia-induced, matrix-specific EMT led to a 1.5-fold increase in glucose uptake (comparing 5 to 25 mM glucose; p < .05; Fig. 3a) that was associated with upregulation of glucose transporter 12 (GLUT12) mRNA (2-fold increase from 5 to 25 mM glucose; p < .05) but not that of GLUT1 or GLUT4 (Fig. 3b and Supplementary Fig. S4). Lactate production was also significantly elevated in cells undergoing EMT (1.5-fold increase from 5 to 25 mM glucose; p < .05) compared to the control cells grown on collagen that were unaffected by the levels of glucose (Fig. 3c); this was accompanied by a parallel increase in the lactate dehydrogenase A (LDHA) mRNA levels (2-fold increase from 5 to 25 mM glucose; p = .01; Fig. 3d), an enzyme which converts pyruvate to lactate. Furthermore, the levels of the lactate monocarboxylate exporter 4 (MCT4) mRNA and that of the lactate importer MCT2 mRNA increased by 1.7-fold (p < .05 and p = .05 respectively) during hyperglycaemia-induced, matrix-specific EMT. (Fig. 3e (i&ii)). Levels of citrate that correspond to activity of the tricarboxylic acid (TCA) cycle increased with rising glucose levels when the cells were grown on collagen but were relatively decreased during hyperglycaemia-induced EMT in the cells grown on fibronectin when compared with cells grown on collagen thereby confirming the more glycolytic phenotype of these cells when exposed to fibronectin (Fig. 3f). Citrate levels increased in a glucose dependent manner on both substrates. Knock down of FASN completely suppressed the EMT associated changes in all of these metabolic parameters to the same degree regardless of the glucose concentration (Fig. 3a–f). To extend our metabolic analysis, we also analysed the mRNA levels of key enzymes related to anabolic pathways essential for macromolecular biosynthesis including phosphoglycerate dehydrogenase (PHGDH), an enzyme involved in the serine biosynthesis pathway, enzymes of the pentose phosphate pathway (glucose-6-phosphate dehydrogenase: G6PD, transketolase: TKT, and transaldolase 1: TALDO1) and hexosamine biosynthetic pathway (glutamine–fructose-6 phosphateransaminase: GFPT1). The mRNA levels of all of these enzymes except PHGDH were unaffected by glucose levels on either matrix (Fig. 3g and Supplementary Fig. S5). PHGDH mRNA expression increased in a glucose-dependent manner in cells cultured on fibronectin (1.5-fold increase from 5 to 25 mM glucose; p < .05) in comparison to those on collagen that were unaffected by the levels of glucose (Fig. 3h). PHGDH and TALDO1 mRNA were significantly suppressed following FASN knockdown on fibronectin to the same degree in all levels of glucose. These results clearly suggest that hyperglycaemia-induced EMT on fibronectin triggers a metabolic switch towards the Warburg effect with increased glucose uptake predominantly via the GLUT12 transporter, increased lactate production along with upregulation of enzymes and transporters for lactate (i.e., LDHA and MCT2 and MCT4), increased serine biosynthesis and a concurrent downregulation of TCA cycle activity.

### 3.4 Antiestrogens fulvestrant and tamoxifen exacerbate hyperglycaemia-induced EMT phenotypic properties irrespective of the metabolic status

**Fig. 4:**
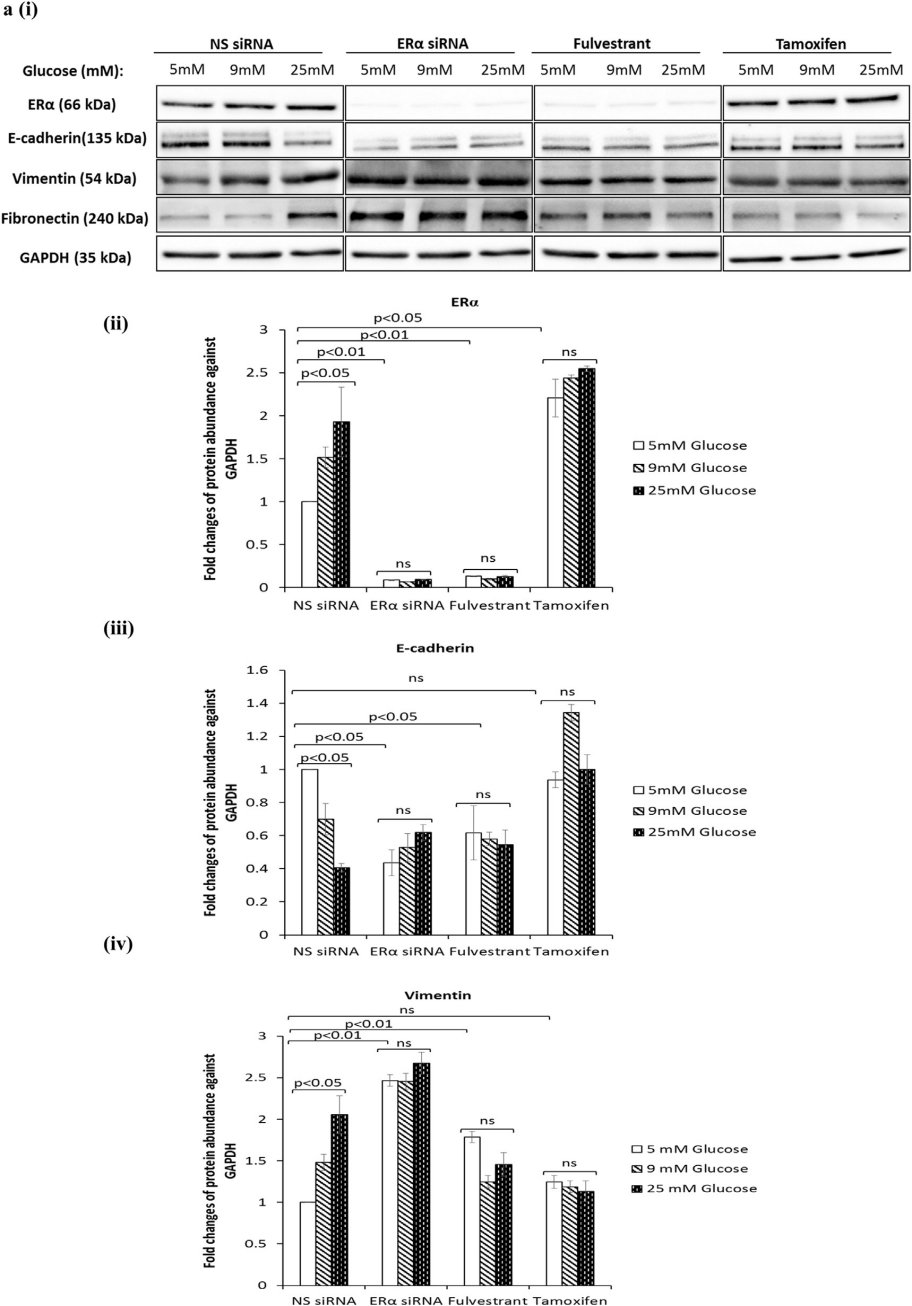

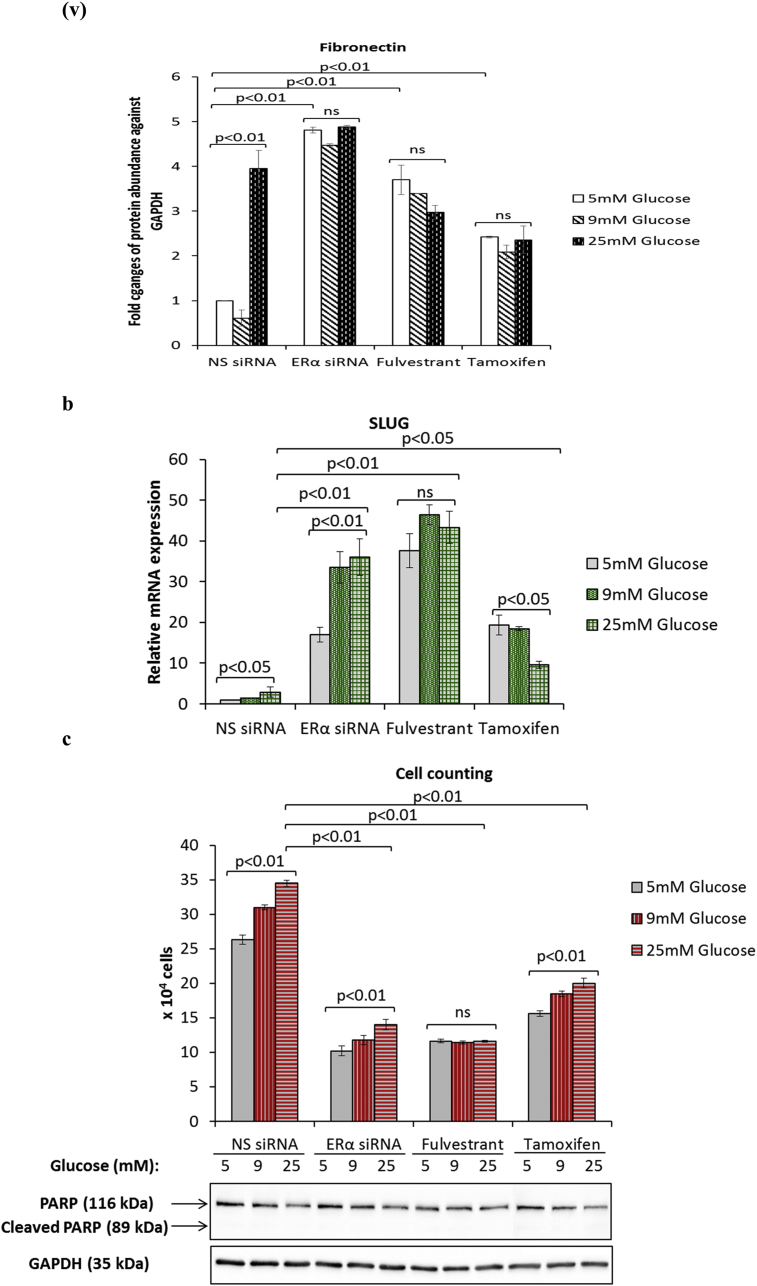

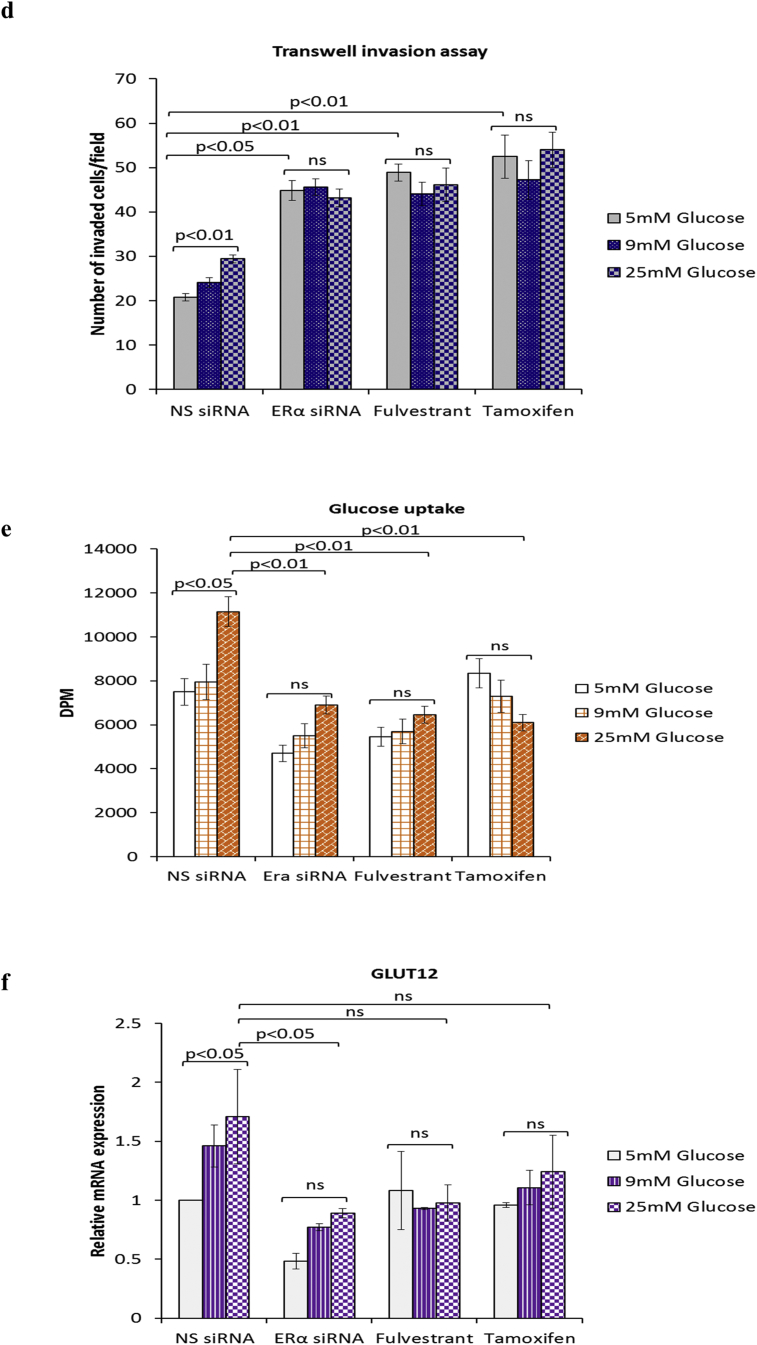

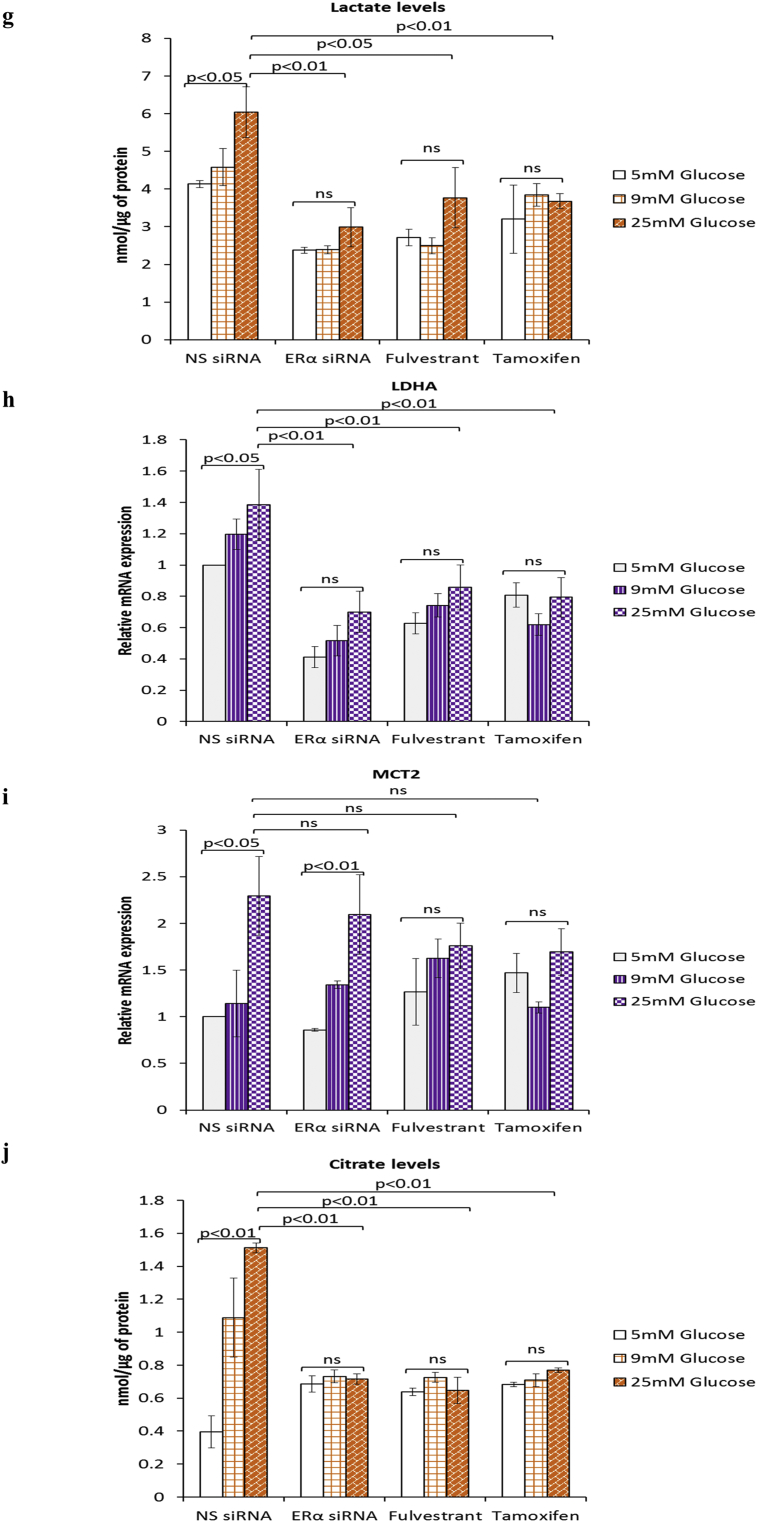
**Inhibition of ERα during hyperglycaemia-induced, matrix-specific EMT results in a more metastatic phenotype irrespective of the glucose concentration**. **(a, i)** Protein expression of EMT markers following downregulation of ERα signalling by siRNA or anti-estrogens fulvestrant (0.1 μM) and tamoxifen (1 μM). **(a, ii)** The densitometry measurements from the western blot are shown. **(b)** Quantification of SLUG mRNA levels as determined by qPCR. **(c**, upper panel**)** Cell growth was assessed as described above. (**c**, lower panel) Western blot detection of PARP cleavage was used as an indicator of apoptosis. **(d)** Cell invasion was measured as described above. **(e**–**i)** Changes to the metabolic parameters following ERα knock down by siRNA and treatment with antiestrogens were assessed as described in the legend of Fig. 3. Results shown are representative of three independent experiments, each performed in triplicate. Data are represented as mean ± SEM. (NS = non-silencing).

ERα is known to regulate and contribute to the Warburg effect in breast cancer cells [23]. Furthermore, we recently showed that ERα functions as a downstream effector of FASN in the context of hyperglycaemia-induced chemoresistance [15]. Given that targeting FASN during hyperglycaemia-induced, matrix-specific EMT was clearly not a good therapeutic approach, as it increased the invasive potential of the cells, we next determined whether targeting downstream ERα signalling would have a similar effect. We blocked ERα signalling using two anti-estrogenic agents, fulvestrant and tamoxifen, that are in clinical use and compared this with silencing the ER using siRNA and assessed any modifications to hyperglycaemia-induced EMT changes. At the doses described previously (0.1 μM fulvestrant and 1 μM tamoxifen) [15], we confirmed that fulvestrant downregulates the ERα and tamoxifen stabilises it as reported in the literature (Fig. 4a (i&ii)) [24]. Fig. 4a (iii) shows that, downregulation of the ERα using either fulvestrant or the siRNA to ERα which mimicked the effects of fulvestrant treatment induced a glucose-independent reduction in E-cadherin and a strong increase in vimentin (Fig. 4a (iv)) and fibronectin (Fig. 4a (v)). A similar trend, although to a much smaller extent was observed in MCF-7 cells treated with tamoxifen (Fig. 4a (iii-v)). Quantification of SLUG mRNA levels revealed a dramatic increase in SLUG expression following treatment with fulvestrant and the ERα siRNA and to a lesser extent by exposure to tamoxifen (ERα siRNA: 13-fold increase at 25 mM glucose; p < .01, fulvestrant: 15-fold increase at 25 mM glucose; p < .01, tamoxifen: 3.3-fold increase at 25 mM glucose; p < .05) (Fig. 4b). We also observed that the effect of glucose varied with each treatment: while there was a glucose-dependent increase in SLUG mRNA with ERα siRNA, with fulvestrant the levels of SLUG mRNA remained the same irrespective of glucose concentration whereas with tamoxifen the effect of glucose appeared to be reversed. Next, we assessed the growth and invasive potential of these cells. Suppression of ERα signalling by siRNA or antiestrogens significantly reduced the growth rate to comparable levels although there still remained a glucose-dependent increase with ERα siRNA and tamoxifen but not with fulvestrant (Fig. 4c, upper panel). The inhibition of cell growth was not a result of apoptosis, as we did not observe an induction of poly(ADP-ribose) polymerase (PARP) cleavage with any treatment (Fig. 4c, lower panel). When analysed for their invasive potential, cells treated with either agent displayed significantly higher and comparable invasive capacity compared to the control cells; although this increased invasive activity was then unaffected by the levels of glucose (e.g. ERα siRNA: 2.2-fold increase at 5 mM glucose; p < .05, fulvestrant: 2.4-fold increase at 5 mM glucose; p < .01, tamoxifen: 2.5-fold increase at 5 mM glucose; p < .01) (Fig. 4d). As shown in Fig. 4e–i, we observed a strong downregulation of all glycolytic components upon treatment with both antiestrogens and the siRNA to ERα. With the exception of MCT2 mRNA whose levels increased in a glucose-dependent manner upon treatment with ERα siRNA other metabolic parameters appeared to follow a similar glucose-independent pattern with all treatments. Taken together, these findings indicate that the current therapeutic strategies using fulvestrant and tamoxifen could result in a more invasive phenotype irrespective of the metabolic status.

### 3.5 Caveolin-1 mediates the pro-invasive properties triggered by FASN or ERα silencing during hyperglycaemia-induced, matrix-specific EMT

**Fig. 5:**
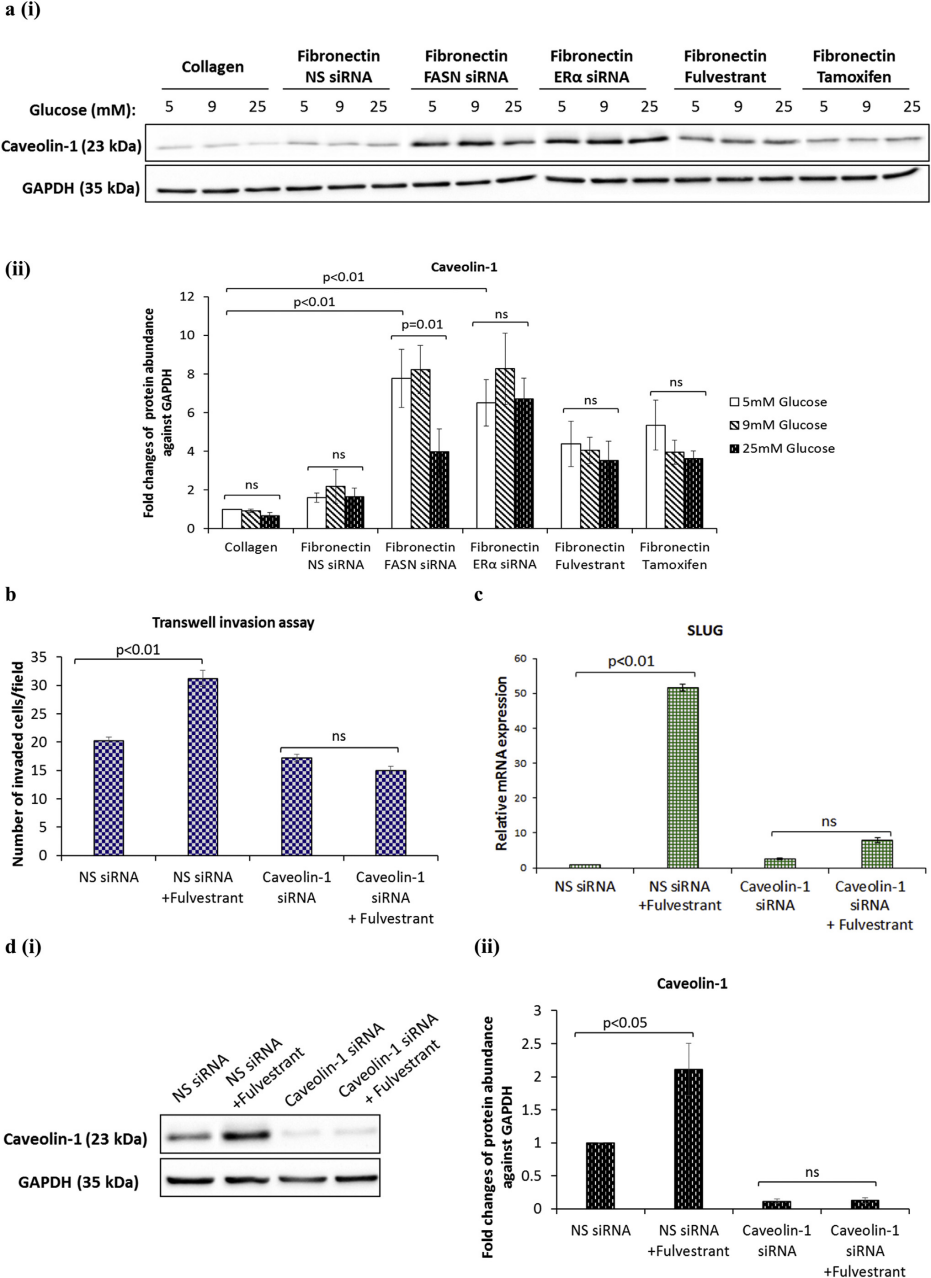
**Silencing of caveolin-1 prevents the increase in cell invasion and SLUG mRNA levels triggered by fulvestrant during hyperglycaemia-induced, matrix-specific EMT**. **(a, i&ii)** Western blot analysis of caveolin-1 protein expression after siRNA-mediated silencing of FASN or ERα and following treatment with antiestrogens fulvestrant and tamoxifen. **(b)** The invasive capacity induced by fulvestrant was assessed in MCF-7 cells with or without caveolin-1 knocked down with siRNA using transwell invasion assay. **(c)** The effect of caveolin-1 knock-down on the fulvestrant-induced SLUG mRNA levels as determined by qPCR. **(d, i&ii)** Caveolin-1 knock-down efficiency was determined by western blotting. Results shown are representative of three independent experiments, each performed in triplicate. Data are represented as mean ± SEM.

**Supplementary Fig. S6:**
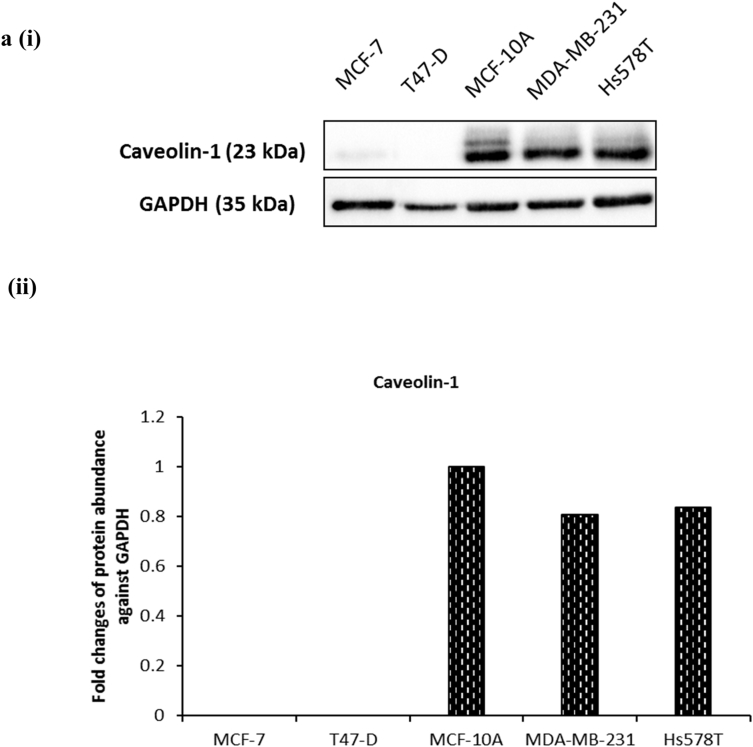
**Differential expression of caveolin-1 in a panel of human breast cell lines**. **(a, i)** The protein levels of caveolin-1 were examined using immunoblotting of the whole cell lysates from the non-malignant MCF-10A cells and various breast cancer cell lines. **(a, ii)** Densitometry was performed to quantify the protein levels of caveolin-1. MCF-10A (non-malignant), MCF-7 and T47-D (non-invasive ERα-positive), MDA-MB-231 and Hs578T (highly invasive ERα-negative) cells.

Caveolin-1 is a lipid raft protein that plays a key role in the regulation of signal transduction pathways and cholesterol transport and recently several studies have shown that caveolin-1 functions as an important regulator of EMT [16,17]. We observed that the protein levels of caveolin-1 were dramatically increased upon FASN or ERα knock down by siRNA and following treatment with antiestrogens fulvestrant and tamoxifen when compared to the non-silencing control: cells displayed the most dramatic increase in caveolin-1 with FASN and ERα silenced (4.8 and 4-fold increase at 5 mM glucose with FASN and ERα knocked down; p < .01 and p < .01 respectively (Fig. 5a (i&ii)). Much higher protein levels of caveolin-1 were also observed in the highly invasive ERα-negative MDA-MB-231 and Hs578T cells when compared to the non-metastatic ERα-positive MCF-7 and T47D cells, indicating a strong correlation between caveolin-1 expression and breast cancer aggressiveness (Supplementary Fig. S6). We therefore assessed whether knock-down of caveolin-1 by specific siRNA is sufficient to prevent the increase in SLUG mRNA levels and cell invasion triggered by fulvestrant; because of the striking phenotypic similarity across different levels of glucose we decided to perform our experiment only under hyperglycaemic conditions. We found that silencing caveolin-1 blocked the ability of fulvestrant to increase cell invasion and SLUG mRNA levels when compared to the non-silencing control (Fig. 5b and c). Effective caveolin-1 silencing is indicated in Fig. 5d (i&ii). Taken together, these results identify caveolin-1 as a critical modulator of the pro-invasive phenotype induced by fulvestrant during hyperglycaemia-induced, matrix-specific EMT.

## 4 Discussion

The role of metabolism in breast cancer has come to the forefront over the last few years owing to the global epidemic in obesity and diabetes and its strong connection with breast cancer. A recently conducted meta-analysis reported a 23% increase in risk of developing breast cancer and a 38% rise in mortality risk among breast cancer patients with diabetes [25]. Another study found that obese women had a 55% greater risk of developing postmenopausal breast cancer [26]. A strong association between metabolic conditions and more aggressive tumour characteristics has also been well-documented [2]. The discovery of the EMT program was a major breakthrough in our understanding of the process of metastasis- the ultimate cause of death in more than 90% of breast cancer patients. However, the impact of metabolic status on EMT and that of EMT on the metabolism of cancer cells has received little attention.

We provide evidence that exposing epithelial breast cancer cells to high levels of glucose and fibronectin, that is associated with an advanced cancer microenvironment, promotes breast cancer progression by driving EMT. Specifically, we report that these microenviromental conditions induce an EMT protein signature, activate SLUG and promote cell growth and invasion. The context-dependent nature of our cell model parallels the well-known Paget's ''seed and soil'' theory, which suggests that cancer cells, referred to as the ''seeds'', are intimately linked to their local microenvironment, called the ''soil'' [27]. We have identified that hyperglycaemia and fibronectin exposure provide a favourable microenvironment that induces EMT together with increases in growth and invasion and further enhanced the Warburg effect. With respect to the latter, we observed that hyperglycaemia-induced, matrix-specific EMT was associated with a significant increase in glucose uptake predominantly via the GLUT12 transporter, higher levels of lactate secretion along with upregulation of the LDHA enzyme and MCT2 and MCT4 lactate carriers and decreased dependency on oxidative phosphorylation (OXPHOS). Similar metabolic changes were recently reported by Kondaveeti et al. in two independent breast cancer cells lines forced to undergo EMT by prolonged mammosphere culture conditions [20]. In that study, the authors also reported substantial increase in glycolytic activity of the cells in response to EMT, as demonstrated by increased glucose uptake, lactate secretion and upregulation of glucose and lactate transporters. In another study, genetic silencing of the respiratory enzyme citrate synthase led to induction of the Warburg effect, EMT and increased metastases in a mouse xenograft model [28]. The crucial contribution of the matrix to EMT has also been documented before. In line with our study, Park and Schwarzbauer demonstrated that exposure to fibronectin but not to matrigel was responsible for transforming growth factor β (TGFβ)-induced EMT in non-transformed MCF10A breast epithelial cells [29].

To understand the mechanism of hyperglycaemia-induced, matrix-specific EMT we focused on our previously defined signalling pathway implicating FASN and the downstream ERα in the regulation of hyperglycaemia-induced chemoresistance [15]. We found that silencing FASN reversed the effects of hyperglycaemia on the levels of EMT markers leading to increased expression of E-cadherin and decreased vimentin and fibronectin. Similar observations were recently made by Jungin et al. in both *in vitro* and *in vivo* models using MCF-7 cells overexpressing mitogen-activated protein kinase 5 (MCF-7-MEK5) where inhibition of FASN signalling with a pharmacological agent cerulenin reversed EMT and promoted mesenchymal to epithelial transition (MET) [30]. We found that inhibiting ERα signalling by either siRNA or using more clinically relevant approaches using the antiestrogens fulvestrant and tamoxifen resulted in a reduction in E-cadherin and strong increases in fibronectin and vimentin irrespective of the levels of glucose. We observed that tamoxifen was the least efficient in this respect, perhaps reflecting its different mode of action.

Most importantly, we show that whilst targeting both FASN and the ERα effectively suppressed cell growth, they also resulted in enhanced invasive capacity with an associated increase in SLUG mRNA levels. FASN has long been recognised as an attractive target for cancer as its inhibition has antiproliferative effects in cancer cells [31] and the antiestrogens fulvestrant and tamoxifen are the most common therapeutic approaches for inhibiting ERα signalling in ER-positive breast cancer patients. However, our findings add to the growing debate over the clinical benefits of these therapeutic drugs. In agreement with our study, Borley and co-workers recently showed that fulvestrant and tamoxifen promote an invasive phenotype in E-cadherin deficient MCF-7 cells by activating Src kinase [32] and others have found that these endocrine agents modulate cell invasion via distinct mechanisms [33]. Furthermore, tamoxifen treatment has been reported to induce the expression of matrix metalloproteinases (MMPs) [34], the well-known ECM-degrading enzymes that participate in tumour invasion [35]. As such, the results reported by us and others shed some light on the current issue of breast cancer recurrence associated with administration of these therapeutic agents [36]. Targeting FASN has also been proposed but likewise needs careful consideration. Stable knock down of FASN in non-obese diabetic/severe combined immunodeficiency (NOD/SCID) mice injected with A549 non-small cell lung cancer cells resulted in increased metastatic spread to lymph nodes, colon, liver and thymus and decreased overall survival [37]. This study is consistent with our findings indicating that silencing FASN correlates with increased invasive potential of the cells despite the reduction in cell growth.

In terms of the metabolic phenotype, we observed a considerable glucose-independent suppression of all glycolytic components upon treatment with both antiestrogens and the siRNA to the ERα and FASN, most likely reflecting the activation of different metabolic pathways. It has in fact been pointed out by a number of investigators that cancer cells within a tumour display a remarkable ability to switch between different metabolic phenotypes depending on the microenviromental challenges and/or treatment modalities to which they are exposed [38], [39]. Even more significant are the findings that such metabolic plasticity is a hallmark of highly aggressive tumours [40]. A study by O'Mahony and colleagues has shown that estrogen can modulate the ability of MCF-7 cells to switch between different metabolic pathways depending on glucose availability [41]. In colorectal cancer cells, stable knock-down of FASN led to a significant decrease in glycolytic capacity [42].

Inhibiting FASN and the ERα was associated with increased invasive capacity and we observed that this was associated with a dramatic up-regulation of caveolin-1. Caveolin-1 is located in plasma membrane invaginations called caveolae: in addition to the presence of caveolin-1, they are also characterised by an abundance of sphingolipids, and cholesterol. Caveolin-1 is considered to play a key role in the regulation of cellular cholesterol homeostasis [43]. Our mechanistic studies identified caveolin-1 as the major driver of the pro-invasive phenotype triggered by targeting FASN or the ERα during hyperglycaemia-induced, matrix-specific EMT. Silencing of caveolin-1 using siRNA blocked the ability of fulvestrant to induce the cell invasion that was accompanied by a reduction in SLUG mRNA levels. Caveolin-1 is highly expressed in invasive breast cancer cells consistent with reports linking caveolin-1 expression with tumour aggressiveness and poorer prognosis [44,45] and more recently caveolin-1 has been shown to play a role in EMT [17,18]. Given the emerging role of caveolin-1 in breast cancer progression, our findings raise the possibility of exploiting caveolin-1 as a therapeutic target to prevent invasion or as a clinical biomarker. As a proof of concept, a previous study showed that inhibition of caveolin-1 signalling by siRNA or caveolin scaffolding peptide in inflammatory breast cancer cells resulted in a reduction in invasive potential in an Akt1-dependent manner [46].

**Fig. 6:**
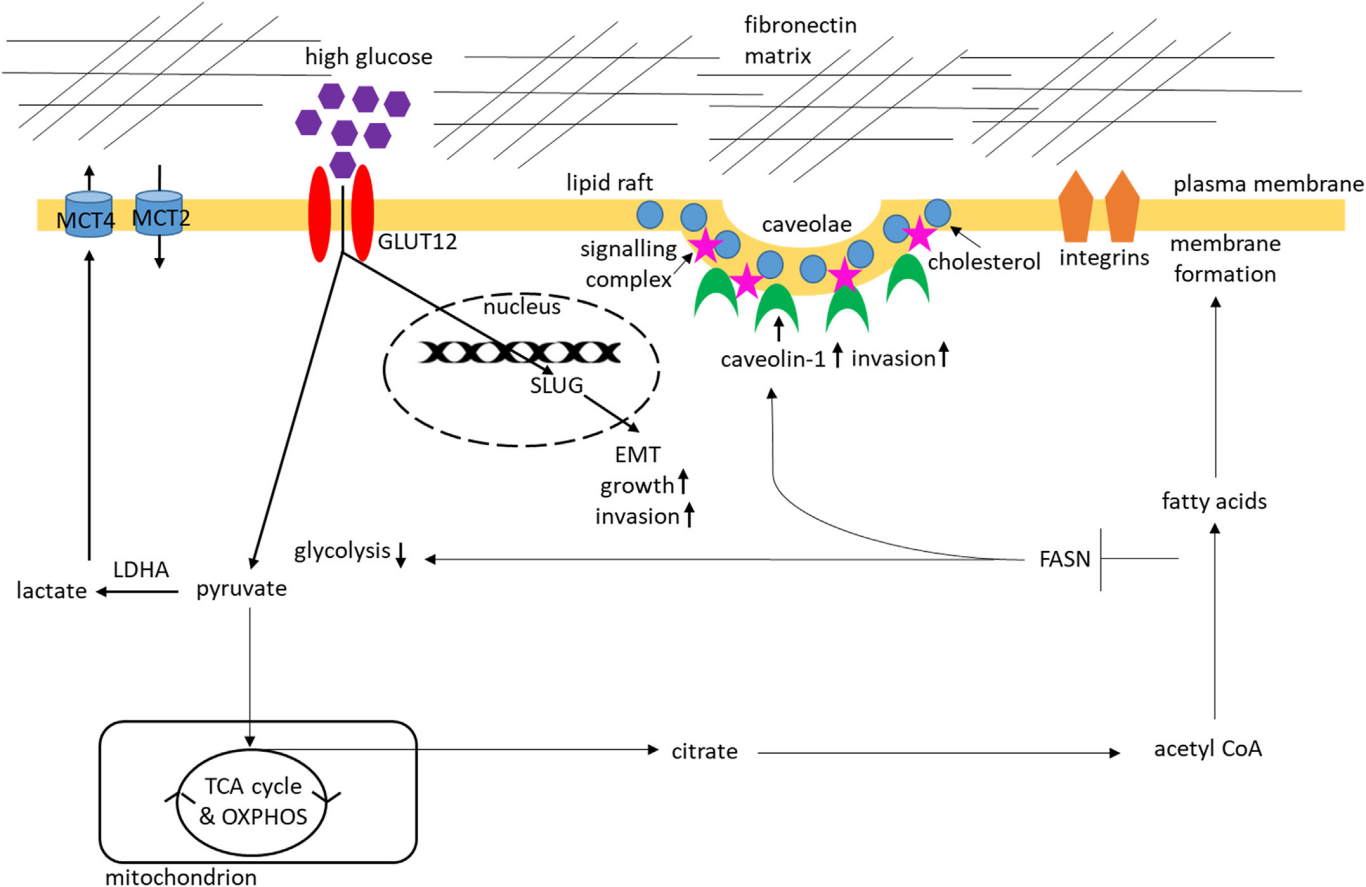
**A schematic diagram illustrating the proposed mechanism of caveolin-1-mediated increase in invasion observed upon silencing FASN/ERα during hyperglycaemia-induced, matrix-specific EMT**. Exposure to hyperglycaemia and fibronectin induces EMT and further enhances the Warburg effect by upregulating glucose uptake, lactate release and specific glycolytic enzymes and transporters. Inhibiting FASN/ERα signalling suppresses the glycolytic phenotype and promotes invasion via upregulation of caveolin-1 in lipid rafts also enriched in cholesterol and various signalling proteins and receptors. Considering the direct involvement of caveolin-1 in mediating cellular cholesterol homeostasis this pro-invasive phenotype is potentially associated with a metabolic shift from glycolysis to cholesterol biosynthetic pathway.

We identified that caveolin-1 mediated the increased invasion when FASN and ERα were inhibited. A recent discovery that the 27-hydroxycholesterol biosynthetic pathway is involved in the regulation of resistance to estrogen deprivation in ERα-positive breast cancer cells [47] may be linked to the way in which caveolin-1 mediates the induction of invasion observed when we targeted the FASN/ERα pathway in an attempt to inhibit hyperglycaemia-induced, matrix-specific EMT in breast cancer epithelial cells. If we assume that this is indeed the case then the acquisition of the pro-invasive phenotype under these experimental conditions might be explained using a very simplified model in which inhibition of ERα and FASN signalling during hyperglycaemia-induced, matrix-specific EMT triggers the upregulation of caveolin-1 in lipid rafts which in turn activates signalling pathways that lead to increased synthesis and possibly accumulation of cholesterol in a population of residual cancer cells surviving the ERα and FASN knock-down (Fig. 6). The hypothesis that these highly invasive cells have used up all available glucose and activate the cholesterol reservoir to maintain this pro-invasive state is very intriguing and so is the concept that these changes might be transient.

Collectively, our study strengthens the clinical connection between metabolic conditions and breast cancer progression by providing a novel mechanistic insight underlying this association. We established that hyperglycaemia and exposure to fibronectin has a major influence on inducing EMT and further promoting the Warburg effect: two processes that are intimately linked. Our data raise concerns regarding targeting the FASN/ERα signalling pathway in ERα-positive breast cancers as inhibiting them enhanced the invasive potential of the cells via a novel caveolin-1-dependent mechanism. If caveolin-1 acts as a pro-invasive switch combined downregulation of caveolin-1 and FASN/ERα signalling might be necessary to effectively suppress cell growth and prevent invasion in a specific subset of ERα-positive breast cancer patients. Translating our *in vitro* data to show the therapeutic potential of this approach *in vivo* would be of great clinical significance.

## Conflict of interest

None.
